# p63 role in breast cancer

**DOI:** 10.18632/aging.101042

**Published:** 2016-10-26

**Authors:** Simone Di Franco, Gianluca Sala, Matilde Todaro

**Affiliations:** Central Laboratory of Advanced Diagnosis and Biomedical Research (CLADIBIOR), University of Palermo, Palermo, Italy

**Keywords:** p63, breast cancer, cancer stem cells, EMT

p63, a transcription factor of the p53 gene family, is known to play a fundamental role in the development of all the stratified squamous epithelia, including breast. The use of two different promoters results in the generation of two different isoforms known as TAp63 and ΔNp63, containing or lacking the trans-activation domain. Each isoform can undergo alternative splicing thus generating different variants, α, β, γ, δ and ε. Since its discovery p63′s physiological role has been controversial with some authors supporting a main role in maintenance of the stem cell compartment and others suggesting that its main role is crucial in driving the differentiation program. It is most likely that indeed p63 exerts both roles with the different variants showing different activities, with the ΔNp63 isoform playing a crucial role in the maintenance of stemness (characterizing stem and progenitor cells), while TAp63 is requested to allow terminal differentiation [1].

Similarly alterations of expression of this gene have been described in cancer where the gene has been suggested to play a role either as a tumor suppressor or an oncogene. Recent work highlights p63 role in breast cancer development, showing that ΔNp63 promotes the breast cancer stem cell (CSC) phenotype by fueling Wnt and Shh pathways activity [2, 3]. Both Wnt and Shh pathways are also crucial for normal mammary stem cell development, and therefore this work further supports a main role for ΔNp63 in maintaining a stem phenotype also in physiological conditions. In our recently published study we have further investigated the role of both p63 isoforms in breast cancer cells, focusing on the mechanisms driving metastatic spreading and acquisition of resistance to conventional anti-tumor therapies [4]. Our findings show for the first time that ΔNp63 positively regulates the PI3K/CD44v6 pathway. We have previously shown that CD44v6 expression in colorectal CSCs activates the Wnt/β-catenin pathway, thus promoting migration and metastasis [7]. Now we show that CD44v6^+^ breast cancer cells present a strong activation of the PI3K pathway, and that its inhibition leads to a reduction in metastatic potential and viability of CD44v6^+^ CSCs. Moreover by transcriptome analysis we show that ΔNp63 increases both tumorigenic and metastatic potential of breast cancer cells by activating the expression of a plethora of genes, including PDGFRB and TWIST1. In line with these results, our *in vivo* experiments show that the ΔNp63 overexpressing cells are localized at the invasive front of the tumor, in proximity of endothelial cells and are therefore more exposed to factors released in the tumor microenvironment. Indeed we show that exposure of breast cancer cells to cytokines such as HGF, SDF-1 and OPN increased ΔNp63 as well as CD44v6 expression levels and activation of the PI3K pathway suggesting that the tumor microenvironment supports a stem like phenotype by inducing ΔNp63 upregulation. We also show that an important consequence of increased levels of ΔNp63 is that BCSCs acquire resistance to aromatase inhibitors and taxanes used in combination with PI3K inhibitors treatments that are currently undergoing clinical trials for the treatment of both luminal and basal breast cancers.

In consideration of all the recent findings, it is clear how the different isoforms of p63 are capable of regulating a large number of target genes involved in different steps of breast cancer progression, including cell proliferation and metastatic capacity. For this reason, it will be crucial in the next future to find a way to selectively target ΔNp63 or its downstream master regulators, such as PI3K or CD44v6. The inhibition of PI3K/CD44v6 axis in combination with current anti-tumor therapies could be a very promising strategy to overcome both primary tumor formation, metastases and relapses.
